# Exploring Exogenous Indole-3-acetic Acid’s Effect on the Growth and Biochemical Profiles of *Synechocystis* sp. PAK13 and *Chlorella variabilis*

**DOI:** 10.3390/molecules28145501

**Published:** 2023-07-19

**Authors:** Wael A. Fathy, Hamada AbdElgawad, Amr H. Hashem, Ehab Essawy, Eman Tawfik, Abdulaziz A. Al-Askar, Mohamed S. Abdelhameed, Ola Hammouda, Khaled N. M. Elsayed

**Affiliations:** 1 Botany and Microbiology Department, Faculty of Science, Beni Suef University, Beni Suef 62511, Egypt; hamada.abdelgawad@uantwerpen.be (H.A.);; 2 Doctoral School of Biology, Faculty of Science and Informatics, University of Szeged, 6720 Szeged, Hungary; 3 Integrated Molecular Plant Physiology Research (IMPRES), Department of Biology, University of Antwerp, BE-2020 Antwerp, Belgium; 4 Botany and Microbiology Department, Faculty of Science, Al-Azhar University, Cairo 11884, Egypt; 5 Biochemistry Division, Chemistry Department, Faculty of Science, Helwan University, Helwan 11795, Egypt; 6 Botany and Microbiology Department, Faculty of Science, Helwan University, Helwan 11795, Egypt; 7 Department of Botany and Microbiology, Faculty of Science, King Saud University, P.O. Box 2455, Riyadh 11451, Saudi Arabia

**Keywords:** abiotic stress, mixotrophic medium, Indole-3-acetic acid, biochemical composition, fatty acid profile

## Abstract

Microalgae have garnered scientific interest for their potential to produce bioactive compounds. However, the large-scale industrial utilization of microalgae faces challenges related to production costs and achieving optimal growth conditions. Thus, this study aimed to investigate the potential role of exogenous indole-3-acetic acid (IAA) application in improving the growth and production of bioactive metabolites in microalgae. To this end, the study employed different concentrations of exogenously administered IAA ranging from 0.36 µM to 5.69 µM to assess its influence on the growth and biochemical composition of *Synechocystis* and *Chlorella*. IAA exposure significantly increased IAA levels in both strains. Consequentially, improved biomass accumulation in parallel with increased total pigment content by approximately eleven-fold in both strains was observed. Furthermore, the application of IAA stimulated the accumulation of primary metabolites. Sugar levels were augmented, providing a carbon source that facilitated amino acid and fatty acid biosynthesis. As a result, amino acid levels were enhanced as well, leading to a 1.55-fold increase in total amino acid content in *Synechocystis* and a 1.42-fold increase in *Chlorella*. Total fatty acids content increased by 1.92-fold in *Synechocystis* and by 2.16-fold in *Chlorella*. Overall, the study demonstrated the effectiveness of exogenously adding IAA as a strategy for enhancing the accumulation of microalgae biomass and biomolecules. These findings contribute to the advancement of microalgae-based technologies, opening new avenues to produce economically important compounds derived from microalgae.

## 1. Introduction

Microalgae and cyanobacteria can produce a wide range of compounds with significant economic, medicinal, and industrial importance. These tiny microorganisms have been attracting significant attention in recent years due to their potential to revolutionize the fields of biofuels, food, medicine, and wastewater treatment [1,2]. In this regard, microalgae are a rich source of valuable compounds, including proteins, lipids, pigments, and carbohydrates, which have significant economic potential [3,4]. For instance, the lipids extracted from microalgae can be converted into biodiesel, which is a clean-burning alternative to traditional diesel fuel [5]. Thus, microalgae are used to produce biofuels, which are renewable energy sources that have the potential to replace fossil fuels [6,7]. Additionally, microalgae are used to produce high-value products, such as astaxanthin, which is a powerful antioxidant with applications in the food, cosmetic, and pharmaceutical industries [8,9]. In the pharmaceutical industry, microalgae and cyanobacteria are used to produce vaccines, antibodies, and other bioactive compounds [10,11]. For instance, economically important products derived from microalgae and cyanobacteria include beta-carotene, phycocyanin, and omega-3 fatty acids [12]. *Spirulina* is a rich source of antioxidants, vitamins, and minerals; in addition, its extract has anti-inflammatory, immunomodulatory, and anticancer properties [13]. Other species such as *Chlorella* and *Dunaliella* are being investigated for their potential health benefits, including improving health, reducing cholesterol levels, and enhancing the immune system [14,15]. Due to their high content of antioxidants and pigments, they can be used in the production of cosmetic products such as anti-aging creams and sunscreens [16,17]. Overall, microalgae and cyanobacteria are promising sources for producing bioactive compounds.

Microalgae and cyanobacteria have immense potential for a wide range of industrial products. However, there are several barriers that prevent their use on an industrial scale. These challenges include high cost of production, as a controlled environment is a necessity and is costly to maintain [18,19]. Achieving optimal growth conditions for microalgae and cyanobacteria is a complex process that requires technical expertise [20]. Additionally, it is challenging to maintain a constant production rate because microalgae and cyanobacteria growth rates depend on a variety of factors, including temperature, light, nutrient availability, and CO_2_ concentration [21,22]. Therefore, further research is required to increase the adaptability and productivity of commercially valuable metabolites while reducing production costs by using effective methods and highly productive strains [23,24].

IAA is a type of plant hormone that is commonly used in plant growth and development studies [25]. IAA’s primary function in microalgae and cyanobacteria is to modulate cell division and proliferation. For instance, it can stimulate cell division and proliferation in these microorganisms, thereby increasing biomass production [26]. For example, IAA stimulates cell division and increases the biomass of microalgae strains such as *Chlorella vulgaris* [27] and *Chlorella pyrenoidosa* [28]. It increases the amount of carotenoid and xanthophyll in the same species [29]. In addition, IAA increases the absorption efficiency of nutrients by microalgae and cyanobacteria, particularly nitrogen and phosphorus [30]. IAA can regulate the responses of microalgae and cyanobacteria subjected to environmental stresses such as nutrient limitation, excessive salinity, heavy metals, and toxic compounds [31,32]. In this regard, IAA activates stress tolerance mechanisms, boosts antioxidant defenses, and enhances overall stress resistance, allowing microorganisms to endure and thrive in challenging environments [32].

Interestingly, cyanobacteria can produce IAA, which promotes growth under normal conditions and provides protection against various stressors [33]. Studies that have examined the growth yield after adding exogenous IAA in quantities critical for hormone production have long established the fact that IAA aids cyanobacteria in growing [34]. Previous studies have indicated that the supplementation of culture media with phytohormones such as IAA and cytokinin can enhance microalgae growth by regulating internal biochemical pathways [35]. Cyanobacteria, however, exhibit distinct responses to IAA depending on their nitrogen fixation capabilities, with synthetic auxins found to enhance dinitrogen fixation and heterocyst frequency in N_2_-fixing cyanobacteria strains [36]. This suggests that IAA plays a significant role in the process of N_2_-fixation by cyanobacteria. Furthermore, the composition of the culture medium affects the growth of Chlorophyta microalgae. An optimal composition of the culture medium enhances microalgae growth and bioactive compound production [37]. It has been found that adding small amounts of plant growth regulators which have the same structures and effects as phytohormones to the culture medium causes biochemical, physiological, and morphological changes [37]. Therefore, the addition of IAA to microalgae culture media is a promising approach for improving biomass production and enhancing biochemical content [38].

This study aims to examine how the addition of exogenous IAA as a plant hormone affects the growth and metabolite profiles, including carbohydrates, amino acids, organic acids, and lipid content, of two strains: the cyanobacterium *Synechocystis* sp. PAK13 and the chlorophyta microalga *Chlorella variabilis*. The research offers a thorough analysis of the unique metabolic responses of these microorganisms, shedding light on their distinct mechanisms for producing biomass and bioactive metabolites.

## 2. Results and Discussion

### 2.1. External IAA Increased IAA Levels in Synechocystis and Chlorella Strains

External exposure to IAA increased the internal IAA content of *Synechocystis* and *Chlorella* (Table 1), whereas the internal IAA content was increased in *Synechocystis* by IAA exposure up to a maximum of 1.42 µM of IAA. Exposure to 0.36 µM of IAA increased the internal IAA content to 0.120 mg/g, which then decreased slightly to 0.107 mg/g and 0.133 mg/g at concentrations of 0.71 and 1.42 µM, respectively. At even higher IAA concentrations (2.84 and 5.69 µM) the internal IAA content increased again, reaching a maximum of 0.236 mg/g. In *Chlorella*, the internal IAA content of the cells in the absence of external IAA is 1.724 mg/g, which increased to 2.25, 3.33, 4.44, 4.71, and 5.81 mg/g after exposure to 0.36, 0.71, 1.42, 2.84, and 5.69 µM of IAA, respectively. The results suggest that both strains can take up and accumulate external IAA, which depends on the concentrations of external IAA exposure as well as the microalgae species. The increase in internal IAA content of *Synechocystis* at low concentrations of external IAA suggests that the cells may be able to use external IAA as a hormone for growth and development. The linear increase in internal IAA content of *Chlorella* with increasing external IAA concentration suggests that the cells can take up and accumulate IAA over a wide range of concentrations. Previous research has shown that IAA exposure increases IAA absorption by microorganisms, including bacteria, cyanobacteria, and microalgae. In this context, the application of exogenous IAA increased the strain’s IAA production, indicating greater IAA absorption [39]. In addition to exogenous IAA uptake, endogenous IAA production is susceptible to manipulation. Prior research indicates that microorganisms such as *Rhodosporidiobolus fluvialis* [40] and cyanobacterial strains such as *Arthrospira platensis* can produce IAA [31]. The study of Meza, et al. [41] proposed that higher IAA production in microorganisms is related to higher accumulations of intracellular phosphate. In this regard, IAA increases the absorption efficiency of nutrients by microalgae and cyanobacteria, particularly phosphorus [30]. Microorganisms can produce more IAA if the growth conditions and medium composition are optimized. The biosynthesis of IAA can contribute to the growth, development, and physiological responses of these microorganisms.

### 2.2. IAA Exposure Improved Growth and Pigments of Synechocystis and Chlorella

**Figure 1:**
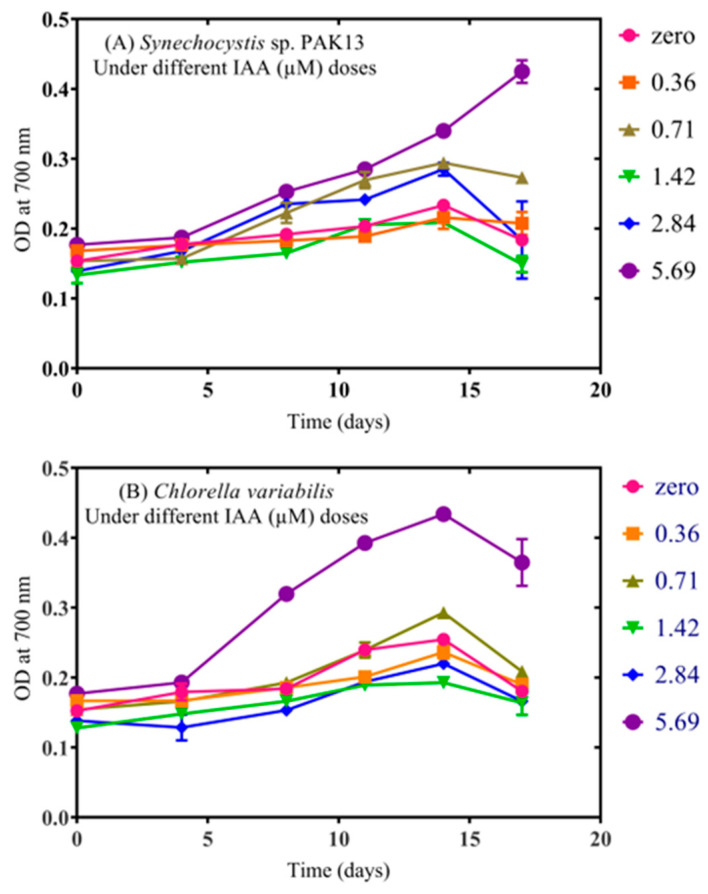
The effect of different external IAA concentrations on the growth curve of (**A**) *Synechocystis* sp. and (**B**) *Chlorella* sp. The figure shows the average of three independent replicates ± SE.

**Figure 2:**
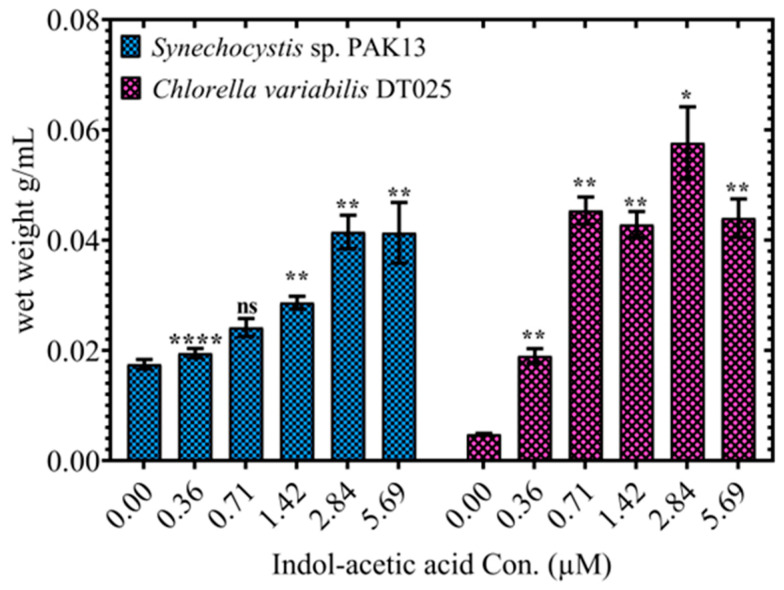
Explaining the effect of different IAA concentrations on wet-weight content in *Synechocystis* sp. and *Chlorella* sp., Data are presented as an average of three independent replicates ± SE. The statistical significances *p* > 0.05, *p* ≤ 0.05, *p* ≤ 0.01 and *p* ≤ 0.0001 are marked by the symbols ns, *, ** and **** respectively.

The growth curves of the two strains displayed were examined to determine the impact of IAA on *Synechocystis* and *Chlorella* (Figure 1). Among all applied IAA concentrations, 0.71 µM of IAA was improved by IAA. Additionally, biomass was steadily increased under IAA treatment, where *Synechocystis* and *Chlorella* produced greater biomass, i.e., 237% and 943%, compared to the control sample (Figure 2). IAA is known to contribute to cell proliferation at low levels by stimulating cell division. However, according to González-Garcinuño, et al. [42] high doses can act as a herbicide and limit growth. We found that growth is affected by different concentrations of external IAA exposure. Biomass accumulation was improved, indicating the positive role of IAA in improving the growth cycle of microalgae. In agreement with Borowitzka, et al. [43], we found that IAA results in a considerable increase in microalgae biomass and cell architectures of *Chlorella zofingiensis*. Moreover, Guldhe, et al. [44] demonstrated that other phytohormones such as gibberellic acid and cytokinin–kinetin increased the biomass percentages of *Chlorella sorokiniana* (35.94% and 37.37%, respectively). Another study found that IAA promoted maximal growth of *S. obliquus* by 1.2-fold when compared to the control [45]. An earlier study showed that adding a mixture of IAA and brassinolide increased *C. vulgaris* growth by four-fold compared to the control [46]. These investigations complement our findings that IAA stimulates cell division and proliferation of microorganisms, resulting in an increase in biomass production [26]. For instance, it increased cell division in *Chlorella vulgaris* [27] and *Chlorella pyrenoidosa* [28].

**Figure 3:**
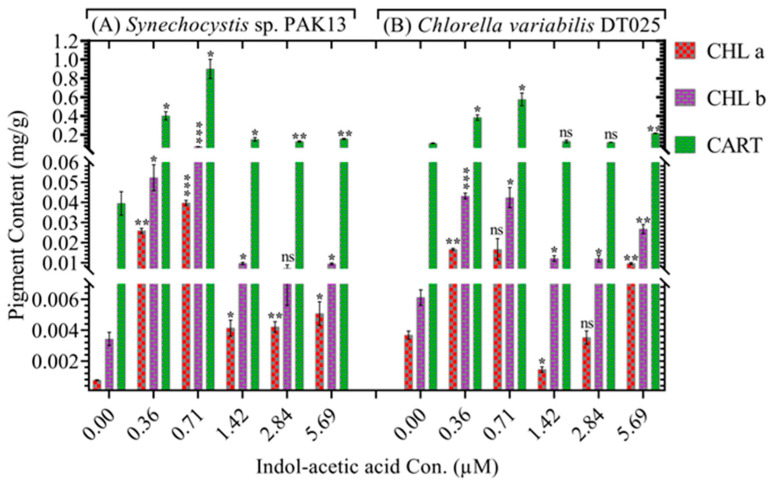
Proving the effect of different IAA concentrations on carotenoids, chlorophyll a, and chlorophyll b content in (**A**) *Synechocystis* sp. and (**B**) *Chlorella* sp. Data are presented as an average of three independent replicates ± SE. The statistical significances *p* > 0.05, *p* ≤ 0.05, *p* ≤ 0.01 and *p* ≤ 0.001 are marked by the symbols ns, *, ** and *** respectively.

To further understand IAA-induced strains biomass accumulation, the content of photosynthetic pigments was measured (Figure 3). Consistent with higher growth, 0.71 µM of IAA increased the chlorophyll level in *Synechocystis* and *Chlorella* 48.75-fold and 4.6-fold, respectively. Similarly, *Synechocystis* and *Chlorella* showed chlorophyll b levels increased by 21.7-fold and seven-fold, respectively. Furthermore, carotenoids were significantly and greatly elevated in *Synechocystis* by 22.7-fold over control and 5.2-fold in *Chlorella*. Thus, IAA treatment boosted microalgae photosynthetic efficiency, allowing them to use more solar energy, which was then transformed into greater biomass and lipids level. Under IAA stress, chlorophyll a, b, and carotenoids were considerably increased in *Synechocystis* and *Chlorella* compared to controls. An increased concentration of carotenoid is a defense mechanism to maintain photosynthetic efficiency by protecting the photosynthesis system [47]. In line with our study, the amount of carotenoid and xanthophyll has been found to increase with IAA exposure in microalgae [29]. The increase in pigments in mesotrophic cultures can at least partially explain the increase in biomass concentration, and corresponds better with an antenna pigment function than with a photoprotector function [48]. Multiple mechanisms can account for the observed increase in photosynthetic pigment content after IAA exposure. It has been reported that IAA stimulates the expression of genes implicated in chlorophyll and carotenoid biosynthesis [49,50]. For instance, it increased the activity of key enzymes such as glycolate oxidase involved in the biosynthesis of pigments [51,52,53]. In addition, IAA exposure increased the efficiency of photosynthetic electron transport and energy conversion, resulting in an increase in pigment synthesis [54]. In general, IAA exposure-induced pigment level increases can be associated with improved photosynthetic efficiency and photosynthetic microorganism growth.

### 2.3. IAA Enhanced Primary Metabolism

#### 2.3.1. Improved Carbohydrates Levels

**Figure 4:**
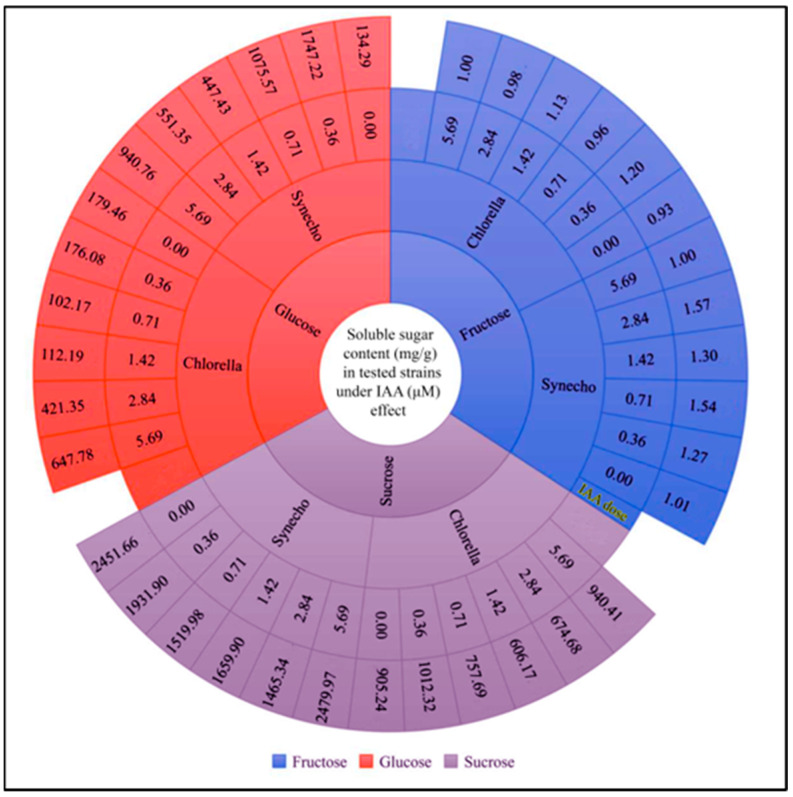
The effects of different IAA (µM) concentrations on fructose, glucose, and sucrose content (mg/g) in *Synechocystis* sp. and *Chlorella* sp. The sunburst shows the average of three independent replicates.

According to our results and a previous study by Lin, et al. [55], external application of IAA improves microalgal growth, photosynthesis, and metabolism. Increased photosynthesis by IAA can induce high carbohydrate accumulation. For instance, treatment with IAA at 0.1 μM increased the concentration of photosynthetic pigments, monosaccharides, and soluble proteins in *C. vulgaris* [56]. Thus, we measured the effect of IAA on carbohydrate levels. Soluble sugars such as glucose were significantly increased in *Synechocystis* with increasing IAA concentrations (Figure 4). *Chlorella* had an eight-fold increase in glucose content compared to the control strain at 0.71 µM IAA. In addition, sucrose levels in *Synechocystis* and *Chlorella* gradually decreased with rising IAA concentrations at ranges between 2.4 mg/g and 1.5 mg/g and 0.9 mg/g to 0.74 mg/g, respectively. On the other hand, fructose content increased slightly with increasing IAA concentrations in both *Synechocystis* and *Chlorella*. Total soluble sugars increased gradually with increasing IAA dose, from 3.03 mg/g for the control strain to 4.45 mg/g at 2.84 μM IAA, before decreasing to 2.03 mg/g at 5.69 μM IAA, as demonstrated in Table 2. On the other hand, the glycogen content increased steadily from 48.44 mg/g at the control level to 72.78 mg/g at 2.84 μM IAA. These results suggest that the optimal IAA concentration for enhancing glycogen production in *Synechocystis* is around 2.84 μM, while higher concentrations may impair glycogen synthesis. The total soluble sugars increased from 3.48 mg/g at the control strain to 4.07 mg/g at 1.42 μM IAA, while the glycogen content increased from 3.54 mg/g at the control to 4.17 mg/g at 1.42 μM IAA. However, at the highest IAA concentration (5.69 μM) both the total soluble sugars and the glycogen content decreased. These findings suggest that an optimal IAA concentration for enhancing the production of total soluble sugars and glycogen in *Chlorella* is around 1.42 μM. IAA has been shown to affect the activity of sugar-metabolizing enzymes in plants, including those involved in photosynthesis, glycolysis, and the TCA cycle [52]. By modulating enzyme activities and gene expression, IAA may enhance microalgal sugar biosynthesis and subsequent metabolism. Moreover, it regulates the allocation of carbon in plants [57]. Because microalgae are known to accumulate lipids, it is conceivable that external IAA could influence carbon allocation in microalgae, thereby favoring sugar metabolism and possibly leading to increased sugar production.

#### 2.3.2. Organic Acids

**Figure 5:**
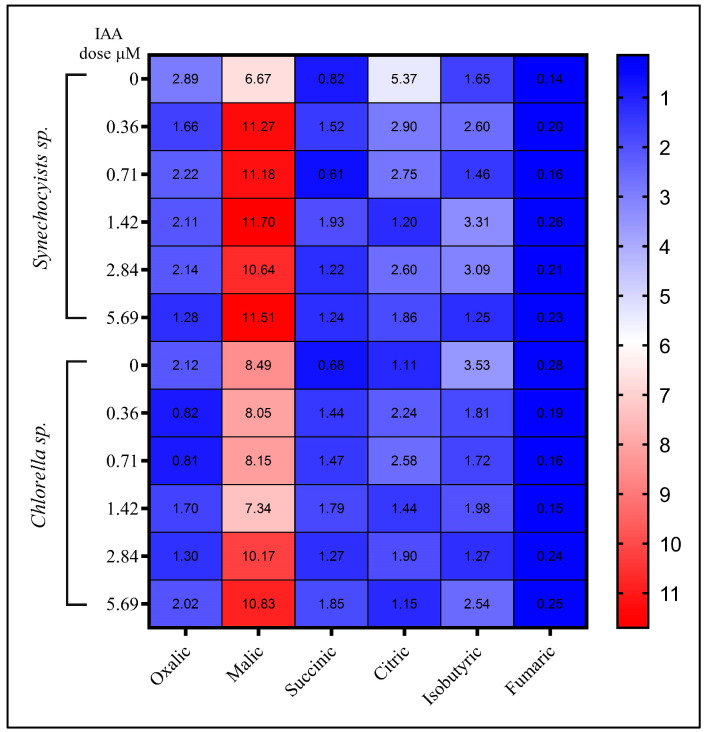
The effect of different IAA concentrations on organic acids content (mg/g) in *Synechocystis* sp. and *Chlorella* sp. The heatmap shows the average of three independent replicates.

The increased availability of carbohydrates and TCA cycle intermediates increases the pool of substrates for energy production and the synthesis of organic, amino, and fatty acids [58]. In the TCA cycle, the complete cycle generates intermediates that serve as precursors for the biosynthesis of organic acids, amino acids, and fatty acids [59]. Organic acids have a wide range of applications and are highly valued in various industries due to their versatility. They play a crucial role in sectors such as the food, energy, chemical, diagnostic, and pharmaceutical industries. In the current study, the changes in sugar levels caused by IAA exposure are likely to have an impact on tricarboxylic acid cycle intermediates such as organic acids (Figure 5), whereas in *Synechocystis* the organic acid profile vary depending on IAA concentrations. In the control strain the dominant organic acid was oxalic acid (2.89 mg/g), followed by citric acid (5.37 mg/g) and malic acid (6.67 mg/g). As the IAA dose increased, the concentration of citric acid decreased while the concentrations of succinic acid, isobutyric acid, and fumaric acid increased. At the highest IAA dose (5.69 μM), the total organic acid concentration was slightly decreased to 17.35 mg/g. On the other hand, in *Chlorella* the dominant organic acid at control conditions was oxalic acid (2.12 mg/g), followed by malic acid (8.49 mg/g) and citric acid (1.11 mg/g). The increase in IAA dose decreased the concentration of oxalic acid, while the concentration of citric acid increased. At the highest IAA dose (5.69 μM), the total organic acid concentration increased to 18.64 mg/g. These results agree with those of Li, et al. [60]; in that study, when various concentrations of IAA were added to poplar seedlings the contents of both GA and malonic acid increased with increasing IAA concentration. An important breakthrough in industrial microbiology has been the ability to produce organic acids from cost-effective raw materials using microbial fermentation. The organic acids in microalgae serve as essential components and building blocks to produce numerous products. For example, citric acid is extensively used in the food industry as an acidulant and flavor enhancer. Similarly, lactic acid finds its application in the production of biodegradable polymers and serves as a pH regulator in food products [61].

#### 2.3.3. Amino Acids Content

**Figure 6:**
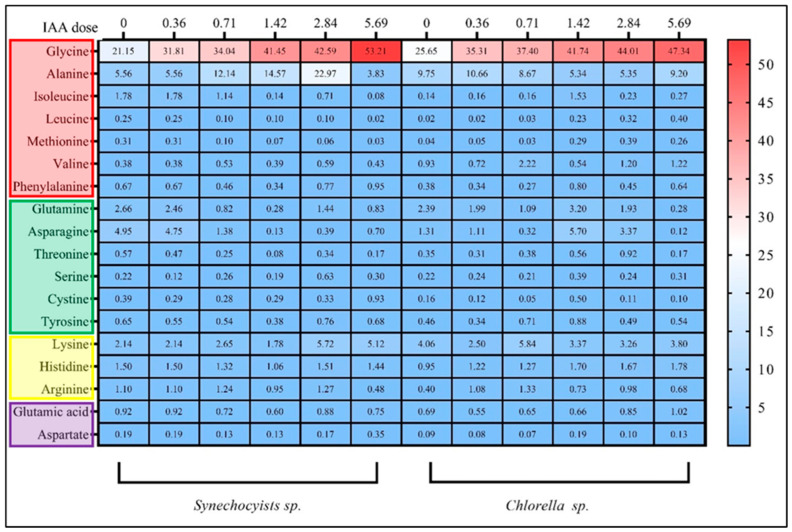
Illustration of the effects of external different IAA concentrations by µM on different types of amino acids content (mg/g) in *Synechocystis* sp. and *Chlorella* sp. The heatmap shows the average of three independent replicates. The red key refers to nonpolar amino acids, green key refers to polar amino acids, the yellow key refers to basic or positively-charged amino acids, and the pink key refers to acidic or negatively charge amino acids.

IAA can modulate the amino acid composition of plants and algal cells when applied externally. Biosynthesis, catabolism, and transport of amino acids can be altered by IAA treatment. It can induce the expression of genes involved in amino acid metabolism and influence the activity of enzymes involved in the synthesis and degradation of amino acids [62]. As a result of the changes in carbohydrates and organic acids, we tracked the changes in amino acid profiles of both strains cultured under varied IAA concentrations. The effectiveness of exogenous IAA on amino acid accumulation, including nonpolar and polar amino acids, was determined to identify the most impacted group, as shown in (Figure 6). For *Synechocystis*, IAA exposure increased most amino acids concentrations, excepting isoleucine and methionine. The highest increase was observed for valine and phenylalanine at the highest IAA dose of 5.69 µM, with a 126% and 43% increase, respectively. Overall, the total concentration of nonpolar amino acids increased by 125% at the highest IAA dose. For *Chlorella*, increasing doses of IAA increased most amino acids, excepting methionine. The highest increase was observed for leucine at the highest IAA dose (5.69 µM, with a 728% increase). The total concentration of nonpolar amino acids increased as well, reaching a maximum of 59.34 mg/g at the highest IAA dose, an increase of 60% compared to the control group. Further *Synechocystis* showed that increasing doses of IAA exposure decreased glutamine, asparagine, threonine, and serine, while cystine and tyrosine decreased at the second-highest dose (2.84 µM). The total polar amino acid content decreased with increasing IAA concentrations. The levels of amino acids in *Chlorella* were more variable, with glutamine, asparagine, and cystine all increasing at the highest dose of IAA and tyrosine increasing at the two highest doses. The total polar amino acid content increased at the highest dose of IAA.

The results for basic or positively charged amino acids and for acidic or negatively charged amino acids showed an increase in the content of basic amino acids, with a 168% increase under the highest IAA concentration treatment (5.69 µM). This increase was mainly due to the increase in arginine content, which increased by 115% at the highest dose. In contrast, the content of acidic amino acids remained relatively stable across all doses, with no significant changes observed. As a result, the total amino acid content increased as the IAA dose increased, while *Chlorella* showed a variable pattern of these levels. At the lowest dose (0.36 µM), the content of basic amino acids decreased while the content of acidic amino acids increased. However, as the IAA dose increased the content of basic amino acids increased as well, with the highest dose resulting in a 76% increase compared to the control. This increase was mainly due to the increase in lysine and arginine content i.e., 78% and 24%, respectively. The total amino acid content increased as the IAA dose increased, with the highest dose resulting in a 43% increase compared to the control. Overall, the results suggest that external IAA exposure can have a significant effect on the amino acid composition of microalgae strains. These effects may be due to differences in the metabolic pathways or biochemical processes between microalgae species. In addition, IAA increases the absorption efficiency of nutrients in microalgae and cyanobacteria, particularly nitrogen and phosphorus, which can consequently improve amino acid metabolism [30].

#### 2.3.4. Fatty Acids Content

The effects of IAA can be expanded to include tricarboxylic acid cycle intermediates such as fatty acids. Moreover, IAA increases the redox status of microalgae, which plays an important role in maintaining oxidative stress in microalgae and increasing lipid synthesis in microalgae under stressful circumstances [63]. As a result, we evaluated individual and total fatty acids (saturated and unsaturated). Total fatty acids increased considerably in *Synechocystis* and *Chlorella* during our study compared to the control sample. To demonstrate these changes, we measured saturated fatty acids (SFA) levels, which showed that SFA levels steadily increased with increasing external IAA doses (Table 3). For *Synechocystis*, exposure to IAA led to an increase in the content of all saturated fatty acids except heptadecanoic acid. Specifically, the highest concentration of IAA (5.69 µM) resulted in a 126% increase in total saturated fatty acids level compared to the control. This increase was mainly due to the significant increase in palmitic acid (97%), stearic acid (28%), and arachidic acid (31%). The concentration of myristic acid was significantly increased (99%) at the highest concentration of IAA. For *Chlorella*, the effect of IAA on fatty acid content was more variable. At low concentrations of IAA (0.36 and 0.71 µM) the fatty acid content did not change significantly compared to the control. However, at higher concentrations of IAA (1.42, 2.84, and 5.69 µM) the content of some fatty acids increased while others decreased. For example, the content of palmitic acid and arachidic acid was significantly increased (89% and 39%, respectively) at the highest concentration of IAA, while the content of heptadecanoic acid and docosanoic acid was significantly decreased at 53% and 36%, respectively.

In addition to unsaturated fatty acids, Table 4 demonstrate that in *Synechocystis* there was an increase in the amounts of all unsaturated fatty acids analyzed at the lowest dose of 0.36 µM, except for palmitoleic acid. The most significant increase was observed in linoleic acid, which increased from 17.78 mg/g to 23.96 mg/g. At the highest dose of 5.69 µM there was a further increase in all unsaturated fatty acids except for heptadecenoic acid. The most significant increase was observed in linolenic acid, which increased from 8.51 mg/g to 31.82 mg/g. The total unsaturated fatty acid content increased from 51.21 mg/g to 92.19 mg/g, while the total fatty acid content increased from 69.92 mg/g to 134.49 mg/g. In *Chlorella*, however, at the lowest dose of 0.36 µM there was a decrease in palmitoleic acid and an increase in all other unsaturated fatty acids. The most significant increase was observed in linoleic acid, which increased from 25.67 mg/g to 24.70 mg/g. At the highest dose of 5.69 µM there was a further increase in the amounts of all unsaturated fatty acids except for oleic acid. The most significant increase was observed in linolenic acid, which increased from 19.36 mg/g to 75.62 mg/g. The total unsaturated fatty acid content increased from 67.33 mg/g to 150.87 mg/g, while the total fatty acid content increased from 86.95 mg/g to 188.21 mg/g. Generally, IAA increased the amount of unsaturated fatty acids in *Synechocystis*, while the effect was more complex in *Chlorella*, with both increases and decreases observed. These results suggest that external IAA can have a significant effect on the fatty acid composition of microalgae.

The majority of existing research has concentrated on microalgae oil and how to induce lipid production using chemical treatments, growing environment alterations, or genetic engineering techniques [64]. Similarly, *Chlorella variabilis* under environmental change accumulates a lipid content of 6.81 × 10^−13^ g/cell, and *Synechocystis* sp. PAK13 has a lipid content of 8.19 × 10^−13^ g/cell [65]. Membrane lipids, one of these enhancements, contain the majority of unsaturated fatty acids, and their principal responsibility is to maintain membrane fluidity in a variety of conditions [66]. Onay [67] reported that the highest dry weight of *Chlorella zofingiensis* was achieved at 80 μM IAA and that the growth cycle can be expected to vary. In another study, when IAA levels were high fatty acid buildup was inhibited in *Scenedesmus* [68]. These findings may have implications for the development of strategies to enhance the production of biofuels and other high-value compounds from microalgae.

## 3. Materials and Methods

### 3.1. Strains and Cultural Conditions

The *Synechocystis* sp. PAK13 and *Chlorella variabilis* DT025 strains utilized in this study were generously provided by the Algal Biotechnology Lab, Faculty of Science, Beni-Suef University, Egypt. These strains were originally isolated from a marine habitat in the Red Sea and cultured using a cost-effective commercial Wuxal medium (WM) supplemented with tap water. WM is a liquid plant fertilizer widely used for fertilization purposes, comprising 8% N, 8% P_2_O_5_, 6% K_2_O, 0.01% B, 0.004% Cu, 0.02% Fe, 0.012% Mn, and 0.004% Zn (Wilhelm Haug GmbH and Co. KG, Ammerbuch, Germany). The culture medium was prepared by adding 800 µL of WM per liter of tap water. To mimic the salinity levels found in the Red Sea habitat, the medium was supplemented with 1 g/L of NaCl. This concentration was selected based on the natural environment from which the strains were originally isolated [22,69]. The growth conditions for the strains were maintained at a temperature of 28 ± 2 °C and a light intensity of 30.4 mol m^−2^s^−1^. The growth medium was supplemented with various concentrations of IAA. The IAA was initially dissolved using NaOH, and concentrations of 0.36, 0.71, 1.42, 2.84, and 5.69 µM were prepared and added to the growth medium. The selection of these IAA concentrations was based on a comprehensive review of the literature, including the studies of Leganés, et al. [36], Zhang, et al. [70] and De-Bashan, et al. [71], as well as preliminary experiments conducted in our laboratory. The chosen concentrations were logarithmically scaled to cover a wide range of doses, allowing for the investigation of potential dose-dependent effects while maintaining a consistent ratio between each concentration.

### 3.2. Growth Parameters

Cell growth was detected by the optical density at 700 nm using a spectrophotometer type (NanBei Instrument^®^, Zhengzhou, China) to calculate the strains growth rate. To determine the wet-weight biomass of the *Synechocystis* and *Chlorella* cultures, we utilized a centrifugation-based approach; specifically, 5 mL of the culture was centrifuged, the pellet was dried in an inverted position on a paper tissue for 5 min at room temperature, then the wet-weight was measured.

### 3.3. Synechocystis and Chlorella Biochemical Profile Estimation

#### 3.3.1. Estimation of IAA

Microalgae were centrifuged for 10 min at 4000× g and the pellets were washed three times at 4 °C with ddH_2_O. Washed pellets (300 mg) were extracted in 2 mL of cooled methanol (80% *v*/*v*). The extracts were collected after centrifugation at 4000× g for 15 min at 4 °C. The extraction was repeated twice, the supernatant was merged, and the volume was increased to 10 mL with 80% pure methanol. To establish liquid chromatography detection, the extract was filtered with a 0.22 μm filter. The contents of IAA were detected by Quaternary gradient ultra-fast liquid chromatography using a Waters Acquity ARC 600-2998 (Waters Corporation®, Milford, MA, USA) equipped with the Symmetry-C18 column (4.6 × 250 mm, 5 μm). Concentrations were calculated according to the calibration curves created with authentic standards.

#### 3.3.2. Estimation of Pigment Content

To determine the levels of photosynthetic pigments in *Synechocystis* and *Chlorella* samples, we adhered to the methodology outlined by Moran, et al. [72]. During the exponential growth phase, a volume of two milliliters was obtained from the culture strains via centrifugation at 13,000 rpm. The resulting pellets were weighted and placed in a covered glass tube, then mixed with 5 mL of 80% ice acetone at 4 °C for 24 h. After centrifugation at 13,000 rpm for 3 min, we collected the supernatants for analysis. The pigments were estimated using the established protocols of Metzner, et al. [73] and Pflanz, et al. [74], while spectrophotometric measurements were taken at 480, 645, and 663 nm.

#### 3.3.3. Estimation of Carbohydrates Content

The levels of soluble carbohydrates were quantitatively estimated in *Synechocystis* and *Chlorella* samples using the method described by Al Jaouni, et al. [75]. During the exponential growth phase, 2 mL of *Synechocystis* and *Chlorella* cultures were extracted in ethanol (80% *v*/*v*) by boiling for 30 S three times and once at room temperature. The derived samples were resuspended in dH_2_O, and the supernatants were kept at 20 °C for further investigation. The concentration of soluble sugars was determined using a Coulter PACE system 5500 and detected using a diode array detector. The concentrations of soluble sugars were calculated using the corresponding standards (glucose, fructose, and sucrose).

#### 3.3.4. Estimation of Organic Acids Content

To extract organic acids from *Synechocystis* and *Chlorella* strains, a mixture of 0.3% (*w*/*v*) butylated hydroxy anisole and 0.1% phosphoric acid was used. The concentrations of citric, succinic, fumaric, and malic acids were estimated using HPLC with a SUPELCOGEL C-610H column and a UV detection system set at 210 nm. The mobile phase was 0.1% (*v*/*v*) phosphoric acid and was eluted at a rate of 0.45 mL/min. To identify and quantify the organic acids, oxalic, malic, succinic, citric, isobutyric, and fumaric acids were used as standards. This method used a LaChrom L-7455 diode array from LaChrom, Tokyo, Japan.

#### 3.3.5. Estimation of Amino Acids Content

The amino acid content of *Synechocystis* and *Chlorella* cultures was analyzed using a modified extraction protocol. Two milliliters of the culture were mixed with 80% (*v*/*v*) aqueous ethanol containing an internal standard (norvaline) to compensate for any loss of amino acids during extraction. The mixture was centrifuged at 20,000 rpm for 20 min and the resulting pellet was resuspended in chloroform. The residue was then re-extracted with HPLC-grade water and the supernatant was combined with the chloroform-suspended pellet. After centrifugation and filtration through Millipore microfilters with 0.2 μm pore sizes, the amino acids were separated using a BEH amide column (2.1 mm × 50 mm) and quantified with a Waters Acquity UPLC-tqd mass spectrometer [76]. A list of amino acid standards, including glycine, alanine, isoleucine, leucine, methionine, valine, phenylalanine, glutamine, asparagine, threonine, serine, cystine, tyrosine, lysine, histidine, arginine, glutamic acid, and aspartate, was used for quantification.

#### 3.3.6. Estimation of Fatty Acids

To extract fatty acids from *Synechocystis* and *Chlorella* biomass, 100 mg of biomass was mixed with 50% aqueous methanol at 25 °C. For identification, Gas Chromatography/Mass Spectrometry (GC/MS) analysis was conducted using an HP-5 MS column (30 µm × 0.25 µm × 0.25 µm) and a Hewlett Packard 6890 GC/MSD 5975 mass spectrometer. Fatty acid identification was performed using the NIST 05 database and the Golm Metabolome Database (http://gmd.mpimp-golm.mpg.de, accessed on 19 December 2022) [77]. A list of fatty acid standards, including myristic, palmitic, heptadecanoic, stearic, arachidic, docosanoic, tricosanoic, pentacosanoic, palmitoleic, heptadecenoic, oleic, linolenic, linoleic and eicosenoic, was used for calibration.

### 3.4. Statistical Analysis

All trials were set up in triplicate using a completely randomized design. The data are reported as (means ± standard error) and visualized using GraphPad Prism 8.4.2 software. One- and Two-Way ANOVA analyses (Tukey Test, *p* ≤ 0.05, 0.01, 0.001, 0.0001) were used for statistical analysis. Each experimental value was compared to the corresponding control value.

## 4. Conclusions

The current research demonstrates the potential use of *Synechocystis* PAK13 and *Chlorella variabilis* DT025 in a mixotrophic medium containing IAA to produce biomass-derived valuable products. Both species were resistant to acute IAA dosages. The results reveal that IAA exposure affects the primary metabolic profiles of the two species in different ways. The addition of a suitable hormone-appropriate concentration can help to meet the high irradiance needs for growth and higher production at high cell density. In these conditions, cell pigments were significantly higher than in autotrophic cultures. These findings indicate that photoautotrophic growth can be replaced with mixotrophic growth. In the presence of IAA, total soluble sugars accumulated significantly at low levels, resulting in increased protein and lipid content. There was a significant link between IAA dose and lipid accumulation in both strains, which was more pronounced in *Chlorella*. The significant lipid output of *Chlorella*, with a high proportion of SFA and USF, shows that it could be exploited as a promising strategy in biodiesel production. Furthermore, the benefits of the conceptual model as well as the economic potential of algal biofuel can be realized with continued industry development and the selection of a high lipid-content algae strain. In this way, the *Chlorella* strain constitutes a compelling argument for a successful industry application strain.

## Figures and Tables

**Table 1:** Showing the effect of external different IAA concentrations on cells IAA hormone in *Synechocystis* sp. and *Chlorella* sp. The data are presented as means ± S.E. (*n* = 3), with different letters indicating a significant difference (*p* < 0.05) between the strain subjected to the IAA dose and the control strain.

Strain	IAA Dose (µM)	IAA Mean ± SE (mg/g)
*Synechocystis* sp.	0	0.103 ± 0.002
	0.36	0.120 ± 0.004 ^a^
	0.71	0.107 ± 0.003 ^a^
	1.42	0.133 ± 0.007 ^b^
	2.84	0.139 ± 0.004 ^b^
	5.69	0.236 ± 0.012 ^b^
*Chlorella variabilis*	0	1.724 ± 0.116
	0.36	2.253 ± 0.055 ^b^
	0.71	3.327 ± 0.127 ^b^
	1.42	4.441 ± 0.131 ^a^
	2.84	4.706 ± 0.053 ^a^
	5.69	5.807 ± 0.136 ^b^

**Table 2:** Estimated total soluble sugars and glycogen content in *Synechocystis* sp. and *Chlorella* sp. grown under different concentrations of IAA for 14 days. The data are presented as means ± S.E. (n = 3), with different letters indicating a significant difference (*p* < 0.05) between the strain subjected to the IAA dose and the control strain.

Strain	IAA Dose (µM)	Total S Sugars (mg/g)	Glycogen Content (mg/g)
		Mean ± SE	Mean ± SE
*Synechocystis* sp.	0	3.03 ± 0.02	48.44 ± 0.36
	0.36	3.66 ± 0.12 ^a^	59.54 ± 0.71 ^a^
	0.71	3.54 ± 0.51 ^a^	62.19 ± 5.23 ^a^
	1.42	4.63 ± 0.27 ^b^	71.25 ± 3.38 ^a^
	2.84	4.45 ± 0.29 ^a^	72.78 ± 4.04 ^b^
	5.69	2.03 ± 0.15 ^b^	36.49 ± 2.14 ^a^
*Chlorella variabilis*	0	3.48 ± 0.18	3.54 ± 0.16
	0.36	3.58 ± 0.04 ^a^	3.90 ± 0.21 ^a^
	0.71	3.40 ± 0.35 ^a^	3.50 ± 0.29 ^b^
	1.42	4.07 ± 0.25 ^b^	4.17 ± 0.21 ^a^
	2.84	2.85 ± 0.30 ^b^	3.10 ± 0.21 ^a^
	5.69	2.62 ± 0.34 ^a^	2.94 ± 0.24 ^a^

**Table 3:** Estimated saturated fatty acids content (mg/g) in *Synechocystis* sp. and *Chlorella* sp. grown under different concentrations of IAA for 14 days. The data are presented as means ± SE. (*n* = 3), with different letters indicating a significant difference (*p* < 0.05) between the strain subjected to the IAA dose and the control strain.

Strain	IAA Dose (µM)	Myristic (C14:0)	Palmitic (C16:0)	Heptadecanoic (C17:0)	Stearic (C18:0)	Arachidic (C20:0)	Docosanoic (C22:0)	Tricosanoic (C23:0)	Pentacosanoic (C25:0)	Sum of Saturated FA ± SE
		Mean ± SE	Mean ± SE	Mean ± SE	Mean ± SE	Mean ± SE	Mean ± SE	Mean ± SE	Mean ± SE	
*Synechocystis* sp.	0	0.332 ± 0.005	15.193 ± 0.333	0.025 ± 0.001	1.393 ± 0.092	1.136 ± 0.013	0.609 ± 0.020	0.020 ± 0.002	0.002 ± 0.0002	18.71
	0.36	0.658 ± 0.006 ^b^	17.458 ± 0.643 ^b^	0.061 ± 0.002 ^b^	1.994 ± 0.104 ^b^	1.228 ± 0.175 ^a^	0.518 ± 0.054 ^a^	0.043 ± 0.004 ^a^	0.004 ± 0.0004 ^a^	21.96
	0.71	0.449 ± 0.002 ^a^	15.801 ± 0.525 ^b^	0.028 ± 0.001 ^b^	1.674 ± 0.090 ^b^	1.282 ± 0.019 ^b^	0.563 ± 0.026 ^a^	0.023 ± 0.003 ^b^	0.002 ± 0.0003 ^a^	19.82
	1.42	0.533 ± 0.002 ^a^	19.611 ± 0.991 ^a^	0.031 ± 0.001 ^a^	2.021 ± 0.110 ^b^	0.878 ± 0.014 ^a^	0.411 ± 0.033 ^a^	0.026 ± 0.003 ^a^	0.003 ± 0.0003 ^a^	23.51
	2.84	0.765 ± 0.004 ^b^	25.423 ± 1.551 ^a^	0.038 ± 0.001 ^a^	1.511 ± 0.093 ^a^	0.812 ± 0.015 ^b^	0.565 ± 0.024 ^b^	0.035 ± 0.001 ^a^	0.003 ± 0.0003 ^b^	29.15
	5.69	1.065 ± 0.003 ^b^	34.647 ± 1.755 ^b^	0.154 ± 0.005 ^a^	4.736 ± 0.253 ^b^	0.786 ± 0.017 ^a^	0.796 ± 0.039 ^a^	0.107 ± 0.013 ^a^	0.008 ± 0.0009 ^a^	42.30
*Chlorella* sp.	0	0.313 ± 0.003	16.254 ± 0.597	0.061 ± 0.003	1.262 ± 0.054	1.225 ± 0.043	0.462 ± 0.077	0.040 ± 0.005	0.004 ± 0.0005	19.62
	0.36	0.630 ± 0.007 ^a^	16.606 ± 0.464 ^b^	0.026 ± 0.001 ^b^	1.227 ± 0.073 ^a^	0.957 ± 0.031 ^a^	0.385 ± 0.020 ^a^	0.020 ± 0.002 ^a^	0.002 ± 0.0002 ^a^	19.85
	0.71	0.751 ± 0.050 ^a^	22.011 ± 2.131 ^b^	0.070 ± 0.007 ^a^	1.718 ± 0.167 ^b^	1.068 ± 0.111 ^a^	0.672 ± 0.043 ^b^	0.035 ± 0.006 ^a^	0.005 ± 0.0009 ^a^	26.33
	1.42	0.478 ± 0.020 ^b^	30.749 ± 0.382 ^a^	0.083 ± 0.006 ^a^	2.371 ± 0.235 ^a^	1.290 ± 0.096 ^b^	0.823 ± 0.049 ^a^	0.055 ± 0.008 ^a^	0.014 ± 0.0010 ^b^	35.86
	2.84	0.383 ± 0.002 ^b^	23.151 ± 0.809 ^a^	0.080 ± 0.003 ^a^	2.242 ± 0.118 ^b^	1.014 ± 0.016 ^a^	0.427 ± 0.020 ^a^	0.055 ± 0.006 ^a^	0.006 ± 0.0006 ^a^	27.36
	5.69	0.262 ± 0.006 ^b^	31.679 ± 3.694 ^a^	0.051 ± 0.001 ^b^	3.254 ± 0.398 ^b^	1.375 ± 0.036 ^b^	0.645 ± 0.090 ^a^	0.060 ± 0.007 ^a^	0.017 ± 0.0035 ^a^	37.34

**Table 4:** Estimated unsaturated fatty acids and total fatty acids content (mg/g) in *Synechocystis* and *Chlorella* grown under different concentrations of IAA for 14 days. The data are presented as means ± SE. (*n* = 3), with different letters indicating a significant difference (*p* < 0.05) between the strain subjected to the IAA dose and the control strain.

Strain	IAA Dose (µM)	Palmitoleic (C16:1)	Heptadecenoic (C17:1)	Oleic (C18:1)	Linolenic (C18:3)	Linoleic (C18:2)	Eicosenoic (C20:1)	Sum of Unsaturated FA ± SE	Total FA
		Mean ± SE	Mean ± SE	Mean ± SE	Mean ± SE	Mean ± SE	Mean ± SE		
*Synechocystis* sp.	0	0.06 ± 0.008	0.138 ± 0.013	27.631 ± 1.424	4.719 ± 0.156	17.780 ± 0.879	0.876 ± 0.038	51.208	69.92
	0.36	0.087 ± 0.009 ^a^	0.165 ± 0.012 ^b^	65.212 ± 1.371 ^a^	7.041 ± 0.459 ^a^	23.963 ± 1.644 ^b^	0.940 ± 0.067 ^a^	97.408	119.37
	0.71	0.075 ± 0.008 ^a^	0.099 ± 0.008 ^a^	35.940 ± 0.942 ^a^	4.645 ± 0.334 ^a^	16.334 ± 1.133 ^b^	0.649 ± 0.044 ^a^	57.742	77.56
	1.42	0.055 ± 0.008 ^a^	0.151 ± 0.013 ^a^	40.946 ± 1.160 ^a^	6.220 ± 0.229 ^a^	20.856 ± 0.522 ^a^	0.813 ± 0.019 ^a^	69.041	92.55
	2.84	0.085 ± 0.004 ^b^	0.169 ± 0.016 ^a^	42.343 ± 0.748 ^b^	7.107 ± 0.545 ^b^	23.923 ± 2.148 ^b^	0.933 ± 0.091 ^a^	74.560	103.71
	5.69	0.280 ± 0.030 ^b^	0.143 ± 0.013 ^a^	50.144 ± 1.826 ^a^	8.508 ± 0.644 ^a^	31.824 ± 2.312 ^b^	1.295 ± 0.093 ^a^	92.193	134.49
*Chlorella* sp.	0	0.093 ± 0.004	0.148 ± 0.015	33.155 ± 1.589	7.239 ± 0.285	25.670 ± 0.615	1.023 ± 0.020	67.328	86.95
	0.36	0.061 ± 0.008 ^a^	0.152 ± 0.016 ^b^	30.122 ± 1.463 ^a^	7.049 ± 0.620 ^a^	24.703 ± 2.038 ^b^	0.981 ± 0.079 ^a^	63.068	82.92
	0.71	0.108 ± 0.003 ^a^	0.150 ± 0.023 ^b^	47.689 ± 1.811 ^a^	6.961 ± 0.58 ^a^	24.297 ± 1.521 ^b^	0.962 ± 0.052 ^a^	80.168	106.50
	1.42	0.138 ± 0.009 ^a^	0.207 ± 0.026 ^a^	38.204 ± 3.362 ^b^	13.143 ± 0.888 ^b^	49.825 ± 2.767 ^b^	2.038 ± 0.104 ^b^	103.556	139.42
	2.84	0.150 ± 0.004 ^a^	0.215 ± 0.017 ^a^	45.462 ± 1.999 ^b^	9.092 ± 0.656 ^a^	30.776 ± 2.435 ^b^	1.204 ± 0.100 ^b^	86.898	114.26
	5.69	0.119 ± 0.017 ^a^	0.264 ± 0.026 ^a^	52.388 ± 0.689 ^a^	19.356 ± 1.383 ^b^	75.619 ± 5.012 ^b^	3.126 ± 0.202 ^b^	150.871	188.21

## Data Availability

Not applicable.
